# Reflections on the Cardiac Surgery Practiced in the1970s when Compared with the Current Practice

**DOI:** 10.21470/1678-9741-2019-0227

**Published:** 2019

**Authors:** Henrique Murad

**Affiliations:** 1 Universidade Federal do Rio de Janeiro, Hospital Universitário Clementino Fraga Filho, Rio de Janeiro, RJ, Brazil.

## INTRODUCTION

I was graduated by the Faculdade de Medicina of the Universidade Federal do Rio de Janeiro (UFRJ) in 1967; I was a general surgery resident at the UFRJ, between 1967 and 1968, and I spent three more years there as a general surgeon. From 1973 to 1976, I did my residency training in cardiothoracic surgery at the University of Illinois (U of I), in Chicago, United States of America (USA). After returning to Brazil, I began my academic career at UFRJ with a high note when I achieved the position of full professor of Cardiothoracic Surgery in 1996. In 2013, I was compulsory retired from the UFRJ, at 70 years of age. After that, I continued working with open-heart surgery in private practice.

During those 46 years of practice, I have observed a tremendous change in cardiac surgery practices, ranging from diagnosis, equipments, and prostheses to surgical techniques and postoperative care.

In this Editorial, I will pinpoint the 10 most important changes that have impressed me in this long period of time.

## DIAGNOSIS

The echocardiogram made a great difference in surgical practice. In 1973, there was only unidimensional echo and most congenital heart disease diagnosis were made by cardiac catheterization. There was only one brain computed tomography (CT) scan in 1973 in Chicago, and the first brain CT scan have arrived in Rio de Janeiro in 1977. Diagnosis of aortic aneurysms could only be made by aortography.

Nowadays, with magnetic resonance imaging, nuclear medicine, transesophageal echo, and so many other new diagnostic gadgets, it is almost impossible to bring a patient with a wrong diagnosis to the operating room.

## EXTRACORPOREAL CIRCULATION

In 1973, at the U of I, we were using disposable bubble oxygenators with a deadly two-hour safety limit for the duration of extracorporeal circulation, and in Brazil, we were still using non-disposable bubble oxygenators, with even shorter safety limits of pump time. Improved pumps, membrane oxygenators, appropriate cannulas, and tubing made a great difference and a four-hour long pump run is not a ticket to heaven anymore.

## MYOCARDIAL PROTECTION

In the 1970s, myocardial protection was made by intermittent cross-clamping, ventricular fibrillation, or local cooling of the heart. Aortic valve replacement was sometimes a nightmare, as we had to rush to end the pump run in less than one hour. Stone heart, an unheard pathology in modern times, was a major cause of anxiety to surgeons at that time. In 1976, Buckberg^[1]^ has changed our practice with the cardioplegia procedure. Since then, we use intermittent blood antegrade cardioplegia.

Braile introduced me to retrograde cardioplegia, which is my favorite way of infusing the cardioplegic solution in aortic surgery. Custodiol or Del Nido^[2]^ cardioplegia have opened new horizons with the possibility of arresting the heart for over 90 minutes without replenishment of the cardioplegic solution.

## POSTOPERATIVE CARE

There were several remarkable changes in this time span. At the beginning, we were not aware of the dangers and were liberal on blood transfusions. The threshold for blood transfusion was a hematocrit of 30%, even if the patient was doing well. Approximately 2/3 of the patients were subjected to blood transfusion at that time. Today, we transfuse about only 20% of our elective cases and accept a hematocrit of 23% without transfusion.

Ventilators have been greatly improved. In the 1970s, they were pressure driven (Bird, Bennett) and oxygen toxicity was a major problem. Currently, most of them are volume driven, with several sensors, and are highly reliable.

Postoperative routine echocardiograms were a major development in postoperative management.

## CONGENITAL HEART SURGERY

In the 1970s and early 1980s, most congenital operations were performed in older children. Neonate surgery was resumed to patent ductus arteriosus ligation, shunts, and coarctation repair. Senning, Mustard, and Jatene operations had a high mortality rate. With the improvement in miniaturized extracorporeal equipment and a better care in the postoperative period, the surgery of the neonate came to a new standard. Tetralogy of Fallot is now corrected during the first year of life; Norwood operation and transplantation came to be a reality and extracorporeal mechanical support has opened a new field in congenital heart surgery. The congenital heart surgery became a subspecialty. Operations developed by Brazilians, like Jatene operation^[3]^ for transposition of the great arteries or the cone operation for Ebstein disease, became available for every congenital heart surgeon.

## MYOCARDIAL REVASCULARIZATION

For decades, coronary artery bypass grafting (CABG) was the most frequent heart surgery performed. I was very fortunate to have spent six months of my residency with Dr Dudley Johnson, in Milwaukee, as his ideas on the subject at that time became full reality as the time went by. He was the first to graft the left anterior descending artery and the first to use multiple grafts. In 1976, he was an advocate of double mammaries, complete revascularization, and endarterectomies^[4]^. The percutaneous use of stents was a major development in myocardial revascularization, but CABG still plays an important role in the care of those patients.

CABG was the most studied operation and over the decades several guidelines were developed for this procedure. We have seen the growing use of off-pump CABG, the use of arterial grafts, and the avoidance of aortic clamps. The use of aspirin, statins, betablockade, and smoking cessation contributed to better short and long-time results after myocardial revascularization.

## VALVE SURGERY

In the 1970s, valve surgery was focused on valve replacement and mitral commissurotomy. Rheumatic heart disease and mitral stenosis were very common. The prostheses available at that time were Starr-Edwards ball valves, Bjork-Shiley and Lillehei-Kaster one-disk tilting valves, the first-generation porcine Hancock valve, and dura mater valves. All those valves were replaced by more effective prostheses, such as the double-disk tilting St Jude valve and the third generation of bovine pericardium valves, like the Carpentier-Edwards Magna Ease.

Surgical mortality was higher than today and surgery on patients over 70 years old was rarely done. Aortic stenosis was operated on in younger patients with bicuspid valves. At that time, we did few operations on degenerative calcified aortic stenosis or degenerative mitral regurgitation, which are so common in patients over 70 years old.

Mitral valve repair was developed by Alain Carpentier and slowly popularized after his monumental paper on “The French Correction”, in 1978^[5]^. In 1992, I went to Paris for Le Club Mitrale to learn how to do mitral valve repair. I can say that mitral valve repair became more frequent in Brazil only after the mid-2000s. Mitral valve chordae replacements with polytetrafluoroethylene suture and annuloplasty rings have changed the way surgeons perform mitral valve repair.

In the past 10 years, major developments contributed to a radical change in the way we perform valve surgery: 1) transcatheter aortic valve replacement, initially done for high-risk patients is now used for intermediate risks and studies have been approved to evaluate its role on low-risk patients as well^[6]^; 2) mitral clips are used for high-risk patients with mitral insufficiency; 3) sutureless aortic valve prosthesis plays an important role in small aortic annulus; 4) a more liberal use of tricuspid valve annuloplasty for patients with secondary tricuspid regurgitation; 5) several catheter-based gadgets are used for mitral annuloplasty, mitral valve replacement, atrial septal defect closure, pulmonary valve replacement, and so on; and 5) aortic valve repair is a field that is expanding very fast after the works of Sievers and el-Khoury^[7]^.

## AORTIC SURGERY

In the 1970s, thoracic aortic operations were very demanding procedures. The grafts we used were tight woven with no bleeding through the mesh, but it would only accept large needles, like the ones of 3-0 prolene, or they were knitted and very porous allowing the use of finer needles, like 5-0 or 6-0 prolene, but they tended to bleed through the mesh. It was very difficult to implant a coronary button of a Bentall procedure with a 5-0 prolene in a tight stiff woven graft. Surgeons tried to overcome this problem by bathing the knitted graft in non-heparinized blood to seal the mesh.

Today we use very porous knitted grafts, which are made watertight after being soaked in gelatin.

Several developments have changed the way we perform aortic surgery procedures: 1) antegrade cerebral perfusion with moderate or deep hypothermia for aortic arch surgery; 2) open distal anastomosis in aortic dissection; 3) biological button Bentall procedure^[8]^; 4) aortic valve remodeling as proposed by Yacoub or aortic valve reimplantation as proposed by David; and 5) antegrade cannulation in aortic dissection.

The most important development in aortic surgery was the use of endoprosthesis. These stent grafts have changed completely our specialty and redirected the surgeons to learn how to do catheter-based procedures.

The surgical mortality in type A acute aortic dissection has changed dramatically over the years. From a dismal around 40% in the 1970s to 18% as published in a recent report of the International Registry of Acute Aortic Dissections (IRAD)^[9]^ and to 5% in selected services.

## HEART TRANSPLANTATION

The first heart transplant, in 1967, done by Christiaan Barnard, in South Africa, had a tremendous impact in cardiac surgery and was almost relegated to oblivion due to tissue rejection. After better control of rejection with cyclosporine and other immunosuppressive drugs, it was reborn in the mid-1980s and good long-term survivals were achieved. Afterwards, in later years, several procedures were conducted to treat heart failure and mechanical circulatory support became a reality^[10]^. Those were patients that in the past were not surgically treated.

## GUIDELINES, DATABASE, AND SURGICAL RISK

As medicine evolved, the number of open-heart procedures increased, and the operative cases became more complex, a demand for guidelines became extremely important. The cardiac surgical and medical specialties got together, and several guidelines were developed to provide standards to be followed by surgeons. The most used guidelines are the ones from the USA (American Heart Association, American College of Cardiology, American Association for Thoracic Surgery, and Society for Thoracic Surgeons [STS]) and Europe (European Society of Cardiology [ESC], European Association for Cardio Thoracic Surgery [EACTS]) on myocardial revascularization, aortic diseases^[11]^, and valvular heart disease. In Brazil, similar guidelines were designed by the Sociedade Brasileira de Cardiologia and the Sociedade Brasileira de Cirurgia Cardiovascular.

Databases were compiled and with the analysis of those data, surgical risk scores were elaborated for several operations performed by cardiac surgeons. There was a leap from an empirical way of executing surgery to a more scientific way of performing one. The most commonly used surgical risk scores for cardiac surgery are the European System for Cardiac Operative Risk Evaluation (EuroSCORE II), from ESC/EACTS, the SYNTAX score, also from ESC/EACTS, and the American STS score. The STS score started to be produced in 1989 and is validated through the analysis of 6,3 millions of cardiac surgical procedures^[12]^.

The open-heart surgery, from diagnosis to the operation and to long-term survival, has improved so much since I began working as a cardiac surgeon in 1973. We now know facts that in the past were mysteries. Residents still work hard, maybe not as hard as my generation did, but they follow the same principles we did: see one, do one, and teach one.

Certain principles have not changed over the decades: our responsibility, our humanity, and our quest for excellence. And I hope they will never do.
